# Biallelic *loss-of-function LACC1/FAMIN* Mutations Presenting as Rheumatoid Factor-Negative Polyarticular Juvenile Idiopathic Arthritis

**DOI:** 10.1038/s41598-019-40874-2

**Published:** 2019-03-14

**Authors:** Raquel Rabionet, Agustín Remesal, Anna Mensa-Vilaró, Sara Murías, Rosa Alcobendas, Eva González-Roca, Estibaliz Ruiz-Ortiz, Jordi Antón, Estibaliz Iglesias, Consuelo Modesto, David Comas, Anna Puig, Oliver Drechsel, Stephan Ossowski, Jordi Yagüe, Rosa Merino, Xavier Estivill, Juan I. Arostegui

**Affiliations:** 1 Centre for Genomic Regulation (CRG), The Barcelona Institute of Science and Technology, Barcelona, Spain; 2 Institut de Recerca Sant Joan de Déu (IRSJD), Esplugues, Spain; 3 Department of Genetics, Microbiology and Statistics, Institut de Biomedicina de la Universitat de Barcelona (IBUB), University of Barcelona, Barcelona, Spain; 4 Department of Pediatric Rheumatology, Hospital La Paz, Madrid, Spain; 5 Department of Immunology, Hospital Clínic-IDIBAPS, Barcelona, Spain; 6 Department of Pediatric Rheumatology, Hospital Sant Joan de Deu, Esplugues, Spain; 7 Department of Pediatric Rheumatology, Hospital Vall d’Hebron, Barcelona, Spain; 8 Departament de Ciències Experimentals i de la Salut, Institute of Evolutionary Biology, CSIC-Universitat Pompeu Fabra, Barcelona, Spain; 9 Genetics and Genomics Program, Sidra Medicine, PObox 26999, Doha, Qatar; 10 Institute of Medical Genetics and Applied Genomics, University of Tübingen, Tübingen, Germany

## Abstract

Juvenile idiopathic arthritis (JIA) is a complex rheumatic disease with both autoimmune and autoinflammatory components. Recently, familial cases of systemic-onset JIA have been attributed to mutations in *LACC1/FAMIN*. We describe three affected siblings from a Moroccan consanguineous family with an early-onset chronic, symmetric and erosive arthritis previously diagnosed as rheumatoid factor (RF)-negative polyarticular JIA. Autozygosity mapping identified four homozygous regions shared by all patients, located in chromosomes 3, 6 (n:2) and 13, containing over 330 genes. Subsequent whole exome sequencing identified two potential candidate variants within these regions (in *FARS2* and *LACC1/FAMIN*). Genotyping of a cohort of healthy Moroccan individuals (n: 352) and bioinformatics analyses finally supported the frameshift c.128_129delGT mutation in the *LACC1/FAMIN* gene, leading to a truncated protein (p.Cys43Tyrfs*6), as the most probable causative gene defect. Additional targeted sequencing studies performed in patients with systemic-onset JIA (n:23) and RF-negative polyarticular JIA (n: 44) revealed no pathogenic *LACC1/FAMIN* mutations. Our findings support the homozygous genotype in the *LACC1*/FAMIN gene as the defect underlying the family here described with a recessively inherited severe inflammatory joint disease. Our evidences provide further support to the involvement of LACC1/FAMIN deficiency in different types of JIA in addition to the initially described systemic-onset JIA.

## Introduction

Juvenile idiopathic arthritis (JIA) refers to an arthritis of unknown origin, starting before the 16^th^ birthday and lasting for at least 6 weeks^1^. It represents the most common pediatric rheumatic condition^2^. Its diagnosis relies on the criteria of the International League of Associations of Rheumatology, defining seven different subtypes: Systemic-onset JIA (SoJIA), oligoarticular, rheumatoid factor (RF)-positive polyarticular, RF-negative polyarticular, enthesitis-related, psoriatic and undifferentiated arthritis^1^. All JIA subtypes are genetically complex disorders that lack a well-defined mode of inheritance, however, as with other complex disorders, there are a small number of cases following Mendelian inheritance. Genome-wide association studies (GWAS) have identified different loci as susceptibility factors, including the MHC and *PTPN22* loci^3–5^. Recently, recessively inherited *LACC1/FAMIN* mutations have been identified in families with monogenic forms of arthritis including SoJIA^6,7^, severe debilitating arthropathy and Crohn’s disease^8^, oligoarticular JIA^7,9^, polyarticular JIA^9^ and enthesitis-related JIA^9^.

We herein describe three siblings born from a consanguineous couple and presenting with RF-negative polyarticular JIA, in which we identified a novel homozygous *LACC1/FAMIN* mutation as the causative defect. These results add novel evidences of the role of this gene in the pathogenesis of a severe form of early-onset inflammatory joint disease and support the clinical diversity of this rare disease. In addition, we present the results of the genetic screening of *LACC1/FAMIN* in 67 JIA patients with different JIA subtypes, performed to assess the role of *LACC1/FAMIN* in sporadic cases of JIA.

## Results

### Clinical description

**Figure 1:**
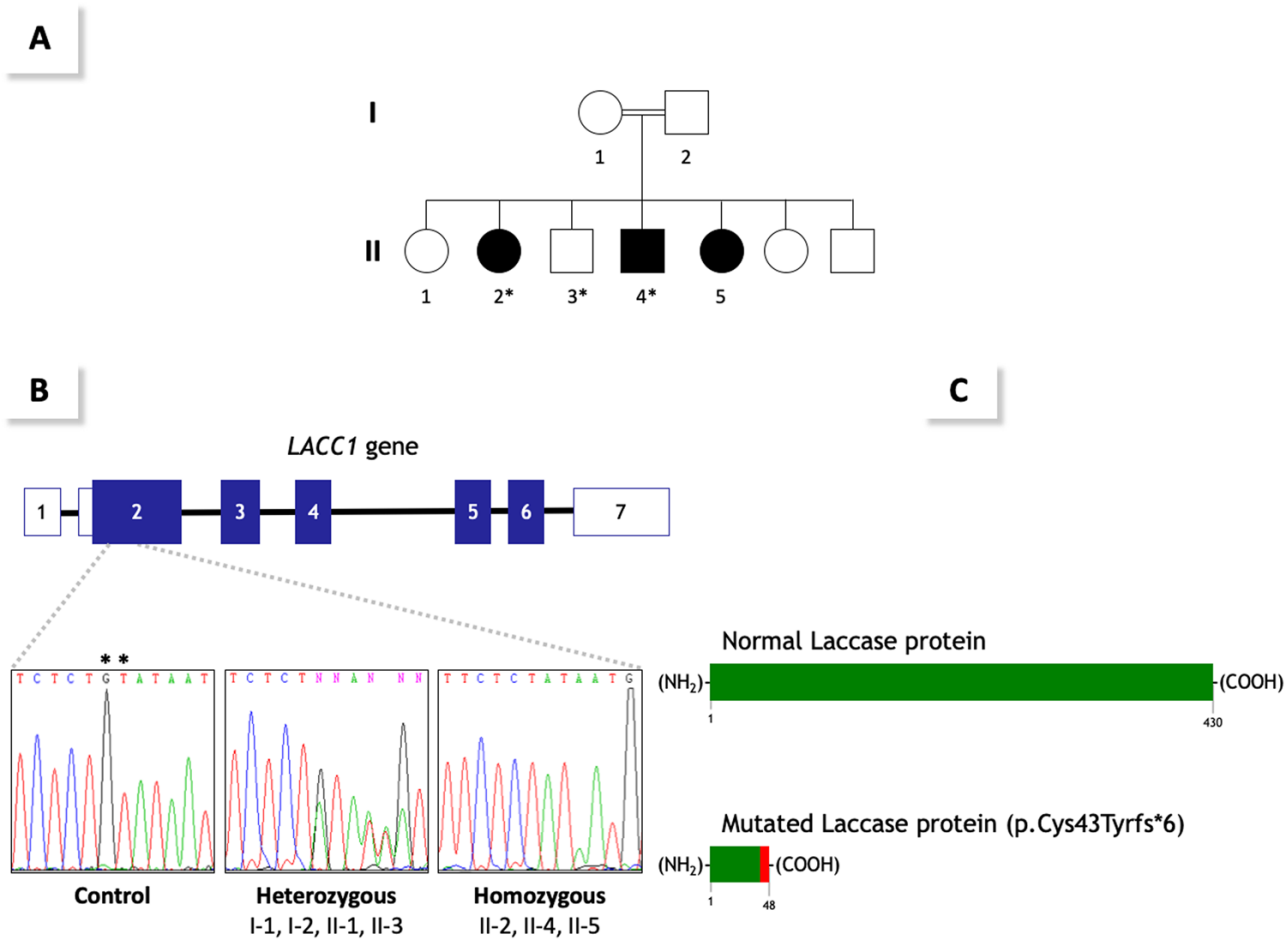
Panel (A). Family’s pedigree. Black filled symbols represent affected individuals, open symbols, unaffected individuals, squares, male individuals, and circles, female individuals. The asterisks indicate individuals evaluated by whole-exome sequencing. Panel (B). (top) Genomic organization of the *LACC1/FAMIN* gene and (bottom) Sanger sense chromatograms from a homozygous wild-type healthy control (left box), heterozygous individuals (middle box), and homozygous mutated patients (right box). The asterisks indicate the two deleted nucleotide positions detected in the patients. Panel (C). (top) Normal structure of laccase protein, and (bottom) predicted structure of the truncated protein encoded by the mutated p.Cys43Tyrfs*6 *LACC1/FAMIN* allele.

We describe three siblings born from a consanguineous Moroccan couple (pedigree in Fig. 1A) with no familial history of autoimmune disease, primary immunodeficiency, metabolic disease or rheumatological disease. All three siblings were afflicted by an early-onset, severe, chronic and symmetric polyarthritis affecting both large and small joints. Fever was detected at disease onset in only one patient, whereas none of the patients presented with skin manifestations at disease onset. In patient II-4, recurrent, self-limited painful erysipelas-like plaques on the legs have appeared in the past two years. Laboratory analyses revealed leukocytosis, thrombocytosis, anemia, marked increases of inflammatory markers, and negative results for RF, anti-nuclear antibodies (ANAs) and HLA-B*27 (See Table 1 and Supplementary Fig. 1 for a detailed description of each patient). These features lead to a proposed diagnosis of RF-negative polyarticular JIA. All patients received both local and systemic treatments, including DMARDs, steroids, anti-TNF, anti-CD20 and anti-IL-6 drugs. All administered treatments were non-effective or provoked only partial responses, with the only exception of the anti-IL-6 tocilizumab that resulted in complete response in Patient II-4 (Table 1).

**Table 1:** Summary of clinical and laboratory data of enrolled patients.

		Patient II-2	Patient II-4	Patient II-5
Gender		Female	Male	Female
Age (yrs)		31	19	17
Disease-onset (yrs)		2	2	3
Muskuloskeletal involvement	Type of arthritis	Polyarthritis	Polyarthritis	Polyarthritis
	Chronic	Yes	Yes	Yes
	Symmetric	Yes	Yes	Yes
	Erosive	Yes	Yes	Yes
	Affected Joints	Temporomandibulars, neck, shoulders, elbows, right MCP, hips, knees	Temporomandibulars, neck, right wrist, right MCP, PIP, DIP, knees, ankles, tarsal, MTP	Temporomandibulars, shoulders, elbows, wrists, MCP, PIP, knees, ankles
	Others	Hip prosthetic replacement at 18 yrs, severe deformity in flexion at hip, primary lymphedema at left leg	Marked failure to thrive (-3SD), marked reduction of ankle mobility, carpitis, tarsitis, astragalus horizontalized, marked muscle atrophy	
Fever		Low-grade fever at disease-onset	No	Yes (at disease-onset)
Skin rash		No	Yes (painful erysipela-like plaque at leg)	No
WBC (normal 3700–11600 cells/ml)*		12880	13200	10880
Platelets (normal 125–350 × 10^3^ cells/ml)*		446 × 10^3^	709 × 10^3^	452 × 10^3^
Haemoglobin (normal 13–17.5 g/dl)*		12.9	9.5	10.6
CRP (normal <5 mg/l)*		71	119	44
ESR (normal <20 mm/h)*		72	92	92
RF		Negative	Negative	Negative
ANAs		Negative	Negative	Negative
HLA-B*27		Negative	Negative	Negative
Synovial Fluid	Cellularity	72180 cells/ml	126000 cells/ml	19900–66700 cells/ml
	PMN	70%	99%	60–94%
Treatments	Not effective	Methotrexate (15 mg/m^2^ q1w)	Methotrexate (15 mg/m^2^ q1w) Etanercept (0.4 mg/kg/twice a week) Infliximab (3 mg/kg/ q8w) Azathioprine (2 mg/Kg q1d)	Systemic and intraarticular steroids Methotrexate (15 mg/m^2^ q1w) Etanercept (0.4 mg/kg/twice a week)
	Partial response	Systemic and intraarticular steroids Infliximab (3 mg/kg/ q8w) Adalimumab (40 mg q2w)	Systemic and intraarticular steroids Rituximab (375 mg/m^2^; 2 doses)	Tocilizumab (8 mg/kg q2w)
	Complete response		Tocilizumab (8 mg/kg q2w)	

Abbreviations: yrs, years; MCP, metacarpophalangeal; PIP, proximal interphalangeal; DIP, distal interphalangeal; MTP, metatarsophalangeal; SD, standard deviation; CRP, C-reactive protein; ESR, erythrocyte sedimentation rate; RF, rheumatoid factor, ANA, anti-nuclear antibodies; PMN, polymorphonuclears. *Laboratory parameters collected at the first visit in the department of Pediatric Rheumatology of the Hospital La Paz.

### Molecular genetics

We postulated an autosomal recessive mode of inheritance for the disease based on the rare phenotype, its presence in individuals of both genders in the same generation, and the presence of familial consanguinity. To identify the underlying gene defect, a combination of genome wide SNP genotyping and WES was performed. SNP genotyping in three affected and one unaffected individuals revealed four different homozygosity regions, located at chromosomes 3, 6 (n: 2) and 13, exclusively shared by all patients. These regions covered approximately 50.61 Mb and contained over 330 genes (Supplementary Table 1). No rare CNVs overlapping with genes were identified in the selected regions. WES was performed in two affected (II-2, II-4) and one unaffected (II-3) siblings. After filtering for novel or rare (minor allele frequency (MAF) <0.005) homozygous variants, two candidate variants within the homozygosity regions were identified: c.506A > T; p.Asp169Val in the *FARS2* gene and c.128_129delGT; p.Cys43Tyrfs*6 in the *LACC1/FAMIN* gene (Table 2). Sanger sequencing in all family members confirmed intrafamilial co-segregation of both variants with the phenotype, following a recessive mode of inheritance. These variants were subsequently genotyped in a control group of healthy Moroccan people. Of the two variants, only the *FARS2* variant was present in the control group (MAF: 0.0014%). Therefore, based on the ACMG classification^10^ we classified the *FARS2* variant as a variant of unknown significance (VUS), as it has not previously been related to disease, it is classified in ClinVar database as a VUS, it is located on an amino acid residue that is not fully conserved through evolution (Supplementary Fig. 2), and the previously *FARS2*-associated phenotypes (spastic paraplegia and combined oxidative phosphorylation deficiency) are not related to arthritis. By contrast, the *LACC1/FAMIN* variant was classified as pathogenic, as it is a null variant (a frameshift 2-bp deletion) that leads to a much shorter open reading frame (p.Cys43Tyrfs*6) (Fig. 1C), it is absent in public databases and in the group of healthy Moroccan controls, and the *LACC1/FAMIN* gene has been linked to various arthritis related phenotypes. Thus, the *LACC1/FAMIN* variant is the most likely causative gene defect for the disorder detected in this family.

**Table 2:** Details of detected gene variants.

Variant	Gene	Refseq^a^ nucleotide and Amino Acid change	Population Genetics								Bioinformatic Analyses			
			1000 Genomes Project (n: 5008 alleles)		ExAC (n: >120000 alleles)		gnomAD (n: >246000 alleles)		Morrocan Healthy Group (n: 704 alleles)		SIFT (Score)	Polyphen-2 (Score)	Mutation Taster	ACMG
			MAF	Het/Hom	MAF	Het/Hom	MAF	Het/Hom	MAF	Het/Hom				
*Chr6:5369309A* > *T*	*FARS2*	c.506A > T p.(Asp169Val)	0.0006	3/0	0.0003	36/0	0.0004	108/0	0.0014	1/0	Del (0)	Pos Dam (0.883)	Dis Caus	VUS
*Chr13 44455248* delGT	*LACC1/FAMIN*	c.128_129delGT p.(Cys43Tyrfs*6)	0	0/0	0	0/0	0	0/0	0	0/0	—	—	Dis Caus	Pathogenic

^a^RefSeq: *FARS2* gene, NM_006567.3; *LACC1/FAMIN* gene, NM_153218.2. Abbreviations: MAF, Minor Allele Frequency; ExAC, Exome Aggregation Consortium; gnomAD, Genome Aggregation Database; SIFT, Sorting Intolerant from Tolerant; ACMG, American College of Medical Genetics and Genomics classification; Het, Heterozygous; Hom, Homozygous; Del, Deleterious; Pos Dam, Possibly Damaging; Dis Caus, Disease Causing; VUS, Variant of Uncertain Significance.

### *LACC1/FAMIN* sequencing in JIA patients

Lately, four articles have described pathogenic or likely pathogenic variants in the *LACC1/FAMIN* gene in patients with different types of JIA^6–9^. To investigate the potential role of this gene in the pathogenesis of sporadic JIA, we sequenced *LACC1/FAMIN* in two different groups of unrelated, sporadic patients of Spanish ancestry with (1) SoJIA (n: 23) and (2) RF-negative polyarticular JIA (n: 44). The sequencing revealed two missense variants, which were not predicted to be pathogenic by functional prediction algorithms. Variant p.Lys38Glu was detected in heterozygosis in one soJIA case, while variant p.Ile254Val was present in heterozygosis in 9 SoJIA and 13 RF-negative polyarticular JIA cases and in homozygosis in two RF-negative polyarticular JIA cases (Table 3). This variant is also present in the Spanish general population. Based on data from the Geuvadis exome variant server (geevs.crg.eu) or the collaborative Spanish variant server (www.csvs.babelomics.org), the populational frequency of the variant is similar to that observed in our dataset (observed MAF in JIA cases 0.194; MAF for Spanish samples 0.2197/0.202; Table 3). This suggests that *LACC1/FAMIN* mutations are not major factors to cause these JIA subtypes.

**Table 3:** Results of *LACC1/FAMIN* genotyping in patients with juvenile idiopathic arthritis (JIA).

JIA subtype	Patients	Nucleotide change^a^	Amino acid change^a^	Genotype	SNP ID	Bioinformatic analyses					
						SIFT (Score)	PolyPhen-2 (Score)	CADD (PHRED)	GEEVS MAF	CSVS MAF	GnomAD MAF
SoJIA	1–13	No changes	No changes								
	14	c.112A > G	p.Lys38Glu	Het	rs34414396	Tolerated (0.31)	Benign (0.0)	15.13	0	0.003	0.0063
	15–23	c.760A > G	p.Ile254Val	Het	rs3764147	Tolerated (1)	Benign (0.001)	16.56	0.2197	0.202	0.2748
RF-negative Pol-JIA	1–29	No changes	No changes								
	30–31	c.760A > G	p.Ile254Val	Hom	rs3764147	Tolerated (1)	Benign (0.001)	16.56	0.2197	0.202	0.2748
	32–44	c.760A > G	p.Ile254Val	Het	rs3764147	Tolerated (1)	Benign (0.001)	16.56	0.2197	0.202	0.2748

^a^NCBI Reference Sequence NM_153218.2. GnomAD MAF: global MAF from the Genome Aggregation Database; GEEVS MAF: MAF in the Spanish dataset of the Geuvadis European Exome data Server; CSVS MAF: MAF from the collaborative Spanish variant server. Abbreviations: SoJIA, systemic-onset JIA; RF, Rheumatoid Factor; Pol-JIA, polyarticular JIA; SIFT, Sorting Intolerant from Tolerant; CADD, Combined Annotation Dependent Depletion.

## Discussion

We describe a consanguineous Moroccan family with a severe joint inflammatory disease that shows a recessive mode of inheritance. Genetic analyses identified the homozygous p.Cys43Tyrfs*6 *LACC1/FAMIN* mutation as the most probable causative gene defect. The *LACC1/FAMIN* gene, previously known as *C13orf31*, encodes for laccase domain containing 1, a member of the blue multicopper oxidases. These enzymes were first identified in plants and fungi, where they catalyze the oxidation of aromatic substrates concomitantly to the reduction of molecular oxygen to water^11^. Previous articles have associated *LACC1/FAMIN* variants with inflammatory diseases, suggesting a potential relationship of human laccase with inflammatory processes^12,13^. Functional studies of wild-type and variant *LACC1/FAMIN* provided a mechanism for its involvement in the inflammatory response as a controller of energy homeostasis in macrophages and show that a reduced or complete loss of function may promote sterile inflammation^14^.

Recent reports have described *LACC1/FAMIN* gene variants in patients with different monogenic JIA subtypes. The first described pathogenic variant in LACC1, p.Cys284Arg, cosegregated with disease in a consanguineous family from Saudi Arabia with a complex phenotype including Crohn’s disease and a severe arthropathy^8^. This same variant was also identified in five additional consanguineous families from Saudi Arabia diagnosed with SoJIA^6^. Subsequently, different homozygous *LACC1/FAMIN* variants have been detected in consanguineous families from various ancestries with different types of JIA^7,9^, including start codon mutations (p.Met1Ile), in-frame deletions (p.Ile330del) and truncating variants (p.Thr276fs*2, p.Arg414Ter). We have identified a novel frameshift truncating *LACC1/FAMIN* mutation in homozygosity in three JIA cases. This variant, p.Cys43Tyrfs*6, generates a shorter open reading frame of only 49 amino acids with a new stop codon in exon 2, which likely leads to nonsense mediated mRNA decay and absence of human laccase in the affected siblings.

All described families with *LACC1/FAMIN* mutations seem to share some clinical features such as the age at disease onset, the hematological and biochemical profiles, and the pattern of joint involvement, including its chronic course, symmetry, and number and type of affected joints. Nevertheless, marked differences in their phenotypes may be also observed. Thus, most of the patients of the families reported by Wakil *et al*., Patel *et al*., and Kallinich *et al*. had fever, cutaneous lesions, serositis, organomegaly or lymphadenopathy, supporting their diagnosis as SoJIA^6–8^. By contrast, most of the patients reported by Karacan *et al*. and the family here described did not present with fever or cutaneous lesions at disease onset. In these patients the joint involvement was the most prominent manifestation at disease onset and showed a chronic and polyarticular course, suggesting the diagnosis of polyarticular JIA^9^. Despite *LACC1/FAMIN* mutations have been repeatedly identified in Mendelian forms of JIA, we failed to detect homozygous or compound heterozygous carriers of rare pathogenic *LACC1* variants in the two groups of Spanish JIA patients analyzed, suggesting that mutations in *LACC1* are not a major cause of JIA, and reinforcing the genetic complexity of this disorder. In fact, although common SNPs in *LACC1/FAMIN* (including variant p.Ile254Val) have been associated to non-systemic JIA in a Swedish cohort^15^, we do not replicate this association in our small dataset, which has a similar carrier frequency to that of the Spanish population obtained from exome sequencing databases. Consequently, all available evidences strongly suggest that biallelic *LACC1/FAMIN* mutations may provoke a severe form of early-onset inflammatory arthritis, and its screening could only benefit those patients with very early-onset JIA.

In summary, our findings describe a novel *LACC1/FAMIN* mutation as the causative defect of a recessively inherited, severe inflammatory joint disease. The clinical features of these patients add novel evidences that the phenotype of this rare genetic disease includes forms of JIA other than SoJIA. The collected data support a relevant role of the *LACC1/FAMIN* gene in inflammatory processes, indicating that further research into its function and its role as a therapeutic target may improve diagnosis and treatment of patients with JIA and other common inflammatory diseases.

## Methods

### Patients

The patients’ data were collected by direct interviews and by the review of their clinical charts. Blood samples were collected for genetic and molecular studies after obtaining written-informed consent from patients or patients’ parents (<18 years), and approval by the ethics committee of Hospital Clinic. All protocols were approved by the Ethics Committee of Hospital Clinic and all methods were performed in accordance with the relevant guidelines and regulations. The control groups included a group of healthy Moroccan individuals (n: 352), and two groups of sporadic Spanish patients with either SoJIA (n: 23) or RF-negative polyarticular JIA (n: 44), which were diagnosed according the criteria of the International League of Associations of Rheumatology. Genetic studies were performed in accordance with the Declaration of Helsinki.

### Homozygosity mapping

DNA was isolated from whole blood using QIAmp DNA Blood Mini Kit (QIAgen, Germany). SNPs were genotyped with HumanCNV370-Duo Beadchip (Illumina Inc, USA), and analyzed for homozygosity mapping using AutoSNPa^16^. Copy number variants (CNVs) detection from SNP genotyping was performed using PennCNV^17^.

### Whole-exome sequencing (WES)

Libraries were prepared with the SureSelect Human All exon V2 kit (Agilent Technologies Inc, USA). Paired-end sequencing was performed on the Illumina Genome Analyzer II platform (Illumina Inc, USA). The sequence reads were aligned to the Human Reference Genome Build hg19 using the BWA software^18^, followed by GATK base quality score recalibration, duplicate marking and local realignment. SNPs and indels were simultaneously called in all samples using the GATK HaplotypeCaller algorithm, applying hard-filtering parameters according to GATK best practices recommendations^19^. Variants were annotated and prioritized using ediva, our in-house pipeline (www.ediva.crg.eu), which provides information on minor allele frequencies from various databases, including the Exome Aggregation Consortium, and Exome Variant Server-NHBLI, variant functional effect prediction scores by SIFT, Polyphen2, MutationAssessor and CADD, and variant conservation scores PhyloP and Gerp++. Fastq files can be accessed through EGA (EGAS00001003510).

### *LACC1/FAMIN* Sanger sequencing

Coding exons of *LACC1/FAMIN* were amplified by PCR, purified with Illustra ExoStar 1-Step kit (GE Healthcare, USA), fluorescence sequenced using ABI BigDye^®^ Terminator v3.1 Cycle Sequencing Kit (Applied Biosystems, USA) and run on an automated ABI 3730XL DNA analyzer.

## Supplementary information

supplementary figures and table
