# Supplementary material for: Biallelic loss-of-function LACC1/FAMIN Mutations Presenting as Rheumatoid Factor-Negative Polyarticular Juvenile Idiopathic Arthritis
Source: Sci Rep. 2019 Mar 14;9:4579. doi: 10.1038/s41598-019-40874-2 (PMC6418186; doi:10.1038/s41598-019-40874-2)
Supplement: Supplementary file 1 — supplementary figures and table [file 41598_2019_40874_MOESM1_ESM.pdf]

Biallelic *loss-of-function LACC1/FAMIN* Mutations Presenting as Rheumatoid Factor-Negative Polyarticular Juvenile Idiopathic Arthritis

Raquel Rabionet [1,2,3,#*], Agustín Remesal [4,#], Anna Mensa-Vilaró [5,6], Sara Murías [4], Rosa Alcobendas [4], Eva Gonzalez-Roca [5], Estibaliz Ruiz-Ortiz [5,6], Jordi Anton [6], Estibaliz Iglesias [6], Consuelo Modesto [7],David Comas [8], Anna Puig [1], Oliver Dreschsel [1], Stephan Ossowski [1,10], Jordi Yagüe [5], Rosa Merino [4], Xavier Estivill [9], Juan I. Arostegui [5*].

Supplementary figures and tables

Legends

Supplementary Figure 1. Panel A. Radiography of Patient II-4 showing a marked narrowing of articular space at right hip and prosthetic left hip replacement performed at the age of 18 years. Panels B and C. Radiographies of Patient II-5 showing carpal bone fusion and subluxation at the right wrist (Panel B) and growth abnormalities and ankylosis affecting both feet (Panel C).

Supplementary Figure 2. Multi-species alignment of *FARS2*-encoded protein. The asterisk indicates the amino acid residue corresponding to the candidate p.Asp169Val gene variant.

Supplementary Table S1. Homozygosity regions shared exclusively by patients detected by genome-wide SNP analysis. The start and end positions from human reference genome build hg19 are shown and the size of the regions is given in bp.

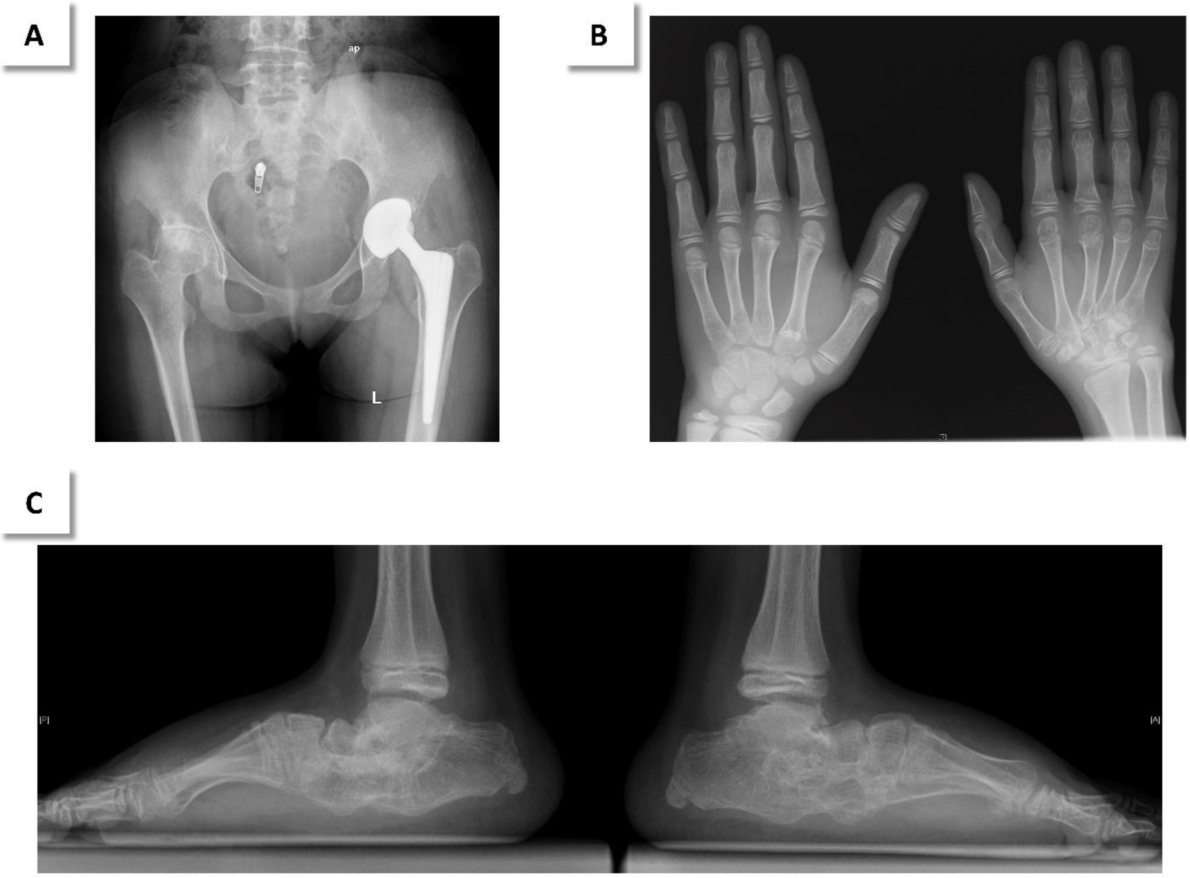

Supplementary Figure 1. Panel A. Radiography of Patient II-4 showing a marked narrowing of articular space at right hip and prosthetic left hip replacement performed at the age of 18 years. Panels B and C. Radiographies of Patient II-5 showing carpal bone fusion and subluxation at the right wrist (Panel B) and growth abnormalities and ankylosis affecting both feet (Panel C).

Supplemental Figure 2

	*
Homo sapiens	SAHQWDLLHAGLDAFLVVGDVYRRD
Pan troglodytes	SAHQWDLLHAGLDAFLVVGDVYRRD
Macaca mulatta	SAHQWDLLHAGLDAFLVVGDVYRRD
Mus musculus	SAHQWDLLHAGLNAFLVVGDVYRRD
Rattus norvegicus	SAHQWDLLHAGLNAFLVVGDVYRRD
Xenopus tropicalis	SAHQWDLMNSGLDAFLAVGDVYRRD
Drosophila melanogaster	TAHQVELISGGLDNFLVVGEVYRRD
Caenorhabditis elegans	SAHQHNLMQSGLDAFLVIGDVYRRD
Takifugu rubripes	LSHPHQNLHSLVVLHLLFIDVYGLV

Supplementary Figure 2. Multi-species alignment of *FARS2*-encoded protein. The asterisk indicates the amino acid residue corresponding to the candidate p.Asp169Val gene variant.

Supplementary Table S1. Homozygosity regions shared exclusively by patients detected by genome-wide SNP analysis. The start and end positions from human reference genome build hg19 are shown and the size of the regions is given in bp.

Chromosome	Start	End	Size	Number of genes
Chr 3	4546671	11571302	7024631	71
Chr 6	5800452	6587594	787142	3
Chr 6	106423900	121477800	15053900	107
Chr 13	42396150	70141036	27744886	149
